# Headache prevalence and its characterization amongst hospital workers in Enugu, South East Nigeria

**DOI:** 10.1186/1746-160X-10-48

**Published:** 2014-11-25

**Authors:** Ikenna Onwuekwe, Tonia Onyeka, Emmanuel Aguwa, Birinus Ezeala-Adikaibe, Oluchi Ekenze, Elias Onuora

**Affiliations:** Neurology Unit, Department of Medicine, University of Nigeria Teaching Hospital, Ituku-Ozalla, Enugu, Nigeria; Department of Anaesthesia/Pain & Palliative Care Unit, University of Nigeria Teaching Hospital, Ituku-Ozalla, PMB 01129, Enugu, Nigeria; Department of Community Medicine, University of Nigeria Teaching Hospital, Ituku-Ozalla, Enugu, Nigeria; Department of Anaesthesia, University of Nigeria Teaching Hospital, Ituku-Ozalla, Enugu, Nigeria

**Keywords:** Headache, Pattern, Health workers, Nigeria

## Abstract

**Background:**

Headaches are probably the commonest neurological complaint worldwide. Amongst workers it contributes significantly to loss of productive time and work efficiency. It is an important cause of disability and reduced quality of life. The prevalence and pattern amongst health workers in Africa has not been extensively studied.

**Objective:**

This epidemiological sampling-based preliminary study examined the frequency and pattern of headache in a population of health workers of a tertiary hospital in Enugu, South East Nigeria.

**Methods:**

Study participants, recruited by balloting, completed a self-administered questionnaire to screen for headache and its associations (defined as headache unrelated to fever and experienced within 6 months prior to the date the questionnaire was administered). Data analysis was by SPSS version 16. Ethical approval was obtained from the Hospital Ethical Review Committee.

**Results:**

One hundred and thirty-three workers aged 18 – 70 years, were evaluated (males 53.4%, n = 71 and females 46.6%, n = 62). Headache was experienced by 88% of workers with primary headaches constituting more than 70% of cases. Females were more affected in both instances. Primary and secondary headaches occurred more in younger and older workers respectively and the association was significant (P <0.05). Headaches were not a significant cause of disability and loss of productivity.

**Conclusion:**

Headaches are very prevalent in hospital workers in Enugu, Nigeria. In older workers screening for underlying causes is indicated. Disability, work absenteeism and loss of productive time are minimal despite the high headache prevalence.

## Introduction

Headache is the commonest neurological disorder in the community with variable intensity, ranging from a trivial nuisance to a severe, disabling, acute or chronic disorder, and may impose a substantial burden on sufferers and on society [1, 2]. It is one of the commonest reasons for visiting the neurology clinics worldwide [3–5], exerting significant burden on its sufferers and impairing daily function especially when accompanied by other symptoms, hence adversely affecting quality of life [6]. According to the World Health Organisation (WHO), 1.7 – 4% of the adult population of the world have headaches on 15 or more days every month [7] and a lifetime prevalence of more than 90% has been attributed to headache disorders in most populations of the world [8].

It is known that Africans have a higher threshold for pain and may not present to the clinic just for an ‘ordinary headache’ [9]. Local experiences show that patients suffering from other chronic neurological disorders present very late to doctors and sometimes never do so [9]. Chronic headaches produce individual and societal burdens, the former referring to its effect on family, social and recreational activities and the latter referring to effects on healthcare cost (direct costs) and work and function (indirect costs), including absenteeism and reduced effectiveness [10].

There is limited data for headache prevalence in Africa. In 2004, the 1-year prevalence of headache from a door-to-door survey of rural south Tanzania was 23.1% (18.8% males and 26.4% females) [11]. Getahu and colleagues in Ethiopia found a 1-year prevalence rate of 73.2% [12]. A 1992 study from Ibadan, South West Nigeria, found the crude life-time prevalence for at least one episode of headache to be 51% [13].

In Nigeria, there is a paucity of data on the national prevalence and burden of chronic headaches [14] despite the fact that it is the commonest presenting neurological disorder in the authors’ environment [1, 3], and therefore the possibility that a big headache problem exists in Nigeria. There are also no known studies of the prevalence and characterization of headache among Nigerian healthcare workers or healthcare workers in South East Nigeria hence the relevance of this study.

### Aim of the study

The aim of this preliminary study was to determine the frequency and pattern of headaches among a population of healthcare workers in a tertiary health institution located in South East Nigeria.

## Methods

**Figure 1:**
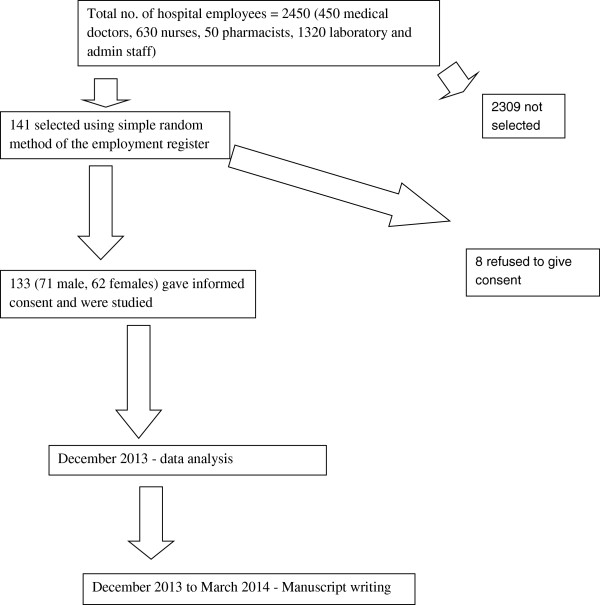
**Flow chart of research activities.**

This was an epidemiological sampling-based study (Figure 1) using a semi-structured questionnaire. The questionnaire was pre-tested in another health facility at Nsukka (a local government area similar to the study area) for content validity. English language was used to reduce cross- cultural misinterpretations and wrong understanding of terms.

The questionnaire was self- administered to all various cadres of health workers in medical unit of University of Nigeria Teaching Hospital, a tertiary health institution located in Enugu, South- East Nigeria, over a 3- month period from September – November 2013, selected by simple random method out of the various units in the hospital e.g. surgical, medical, laboratory, physiotherapy, nutrition, administrative, laundry, transport, security, and medical record. Within these are various cadres of hospital staff: physicians, nurses, pharmacists and cleaners. Out of a total of 141 only 133 gave consent and hence were studied, giving a response rate of 94.3%. To ascertain the overall prevalence of headache, subjects were asked if they have ever had a headache within the previous six months and to note any association. They were to rate the severity of headache based on a scale of mild, moderate and severe. The impact of these severe headaches on the daily activity and the number of days they occur in a month were recorded. The character of the pain, location, duration, and the total numbers of times in the 6 months preceding the date of administering the questionnaire were also noted.

Statistical Package for Social Sciences version 16 was used in statistical analysis. Comparison of multiplex groups was carried out with One Way ANOVA test. On the other hand comparison of two distinct groups was carried out with student t test. Chi-square test (and/or Fisher’s exact test) was used in analysis of categorical variables. The results were revealed as mean ± SD. P value <0.05 was interpreted as statistically meaningful. Ethical approval was obtained from the hospital ethics committee.

## Results

Of the 2,450 hospital employees (450 medical doctors, 630 nurses, 50 pharmacists, and 1320 laboratory and administrative staff), 141 were selected using simple random method from the employment register and eventually only 133 health workers (71 males and 62 females) gave informed consent and were studied (response rate 94.3%). More of the respondents were males (53.4%) and most were within the 25 - 34 years age group (46.6%). Most of the workers had worked for only ≤5 years (72.9%). Table 1 illustrates.

**Table 1:** **Demographic distribution and work experience of health workers**

Variable	Frequency	Percent
***Sex***		
Male	71	53.4
Female	62	46.6
Total	133	100.0
***Age Group***		
15 – 24	13	9.8
25 – 34	62	46.6
35 – 44	36	27.1
45 – 54	15	11.3
55 – 64	6	4.5
65 and above	1	0.7
Total	133	100.0
***Number of years worked***		
1 – 5	97	72.9
6 – 10	18	13.5
11 – 15	7	5.3
16 – 20	4	3.0
21 – 25	5	3.8
26 – 30	2	1.5
Total	133	100.0

The prevalence of headache in the past 6 months was 88.0% (among males the prevalence was 87.3% while in females it was 88.7%). There was no significant difference observed between the sexes (p = 0.806). In both sexes, primary headaches were more prevalent (71.0% in males and 76.4% in females). There was also no significant difference in the prevalence of the primary headaches among the sexes (p = 0.509). See Table 2.

**Table 2:** **Prevalence of headache among the health workers**

General prevalence of headache in the past 6 months		
Variables	Frequency	Percent
Headache present	117	88.0
Headache absent	16	12.0
**Sex prevalence of headache**		
	Male (%)	Female (%)
Headache present	62 (87.3)	55 (88.7)
Headache absent	9 (12.7)	7 (11.3)
Total	71 (100.0)	62 (100.0)
χ^2^ = 0.060; P value = 0.806		
Type of headache		
Primary	44 (71.0)	42 (76.4)
*Secondary	18 (29.0)	13 (23.6)
Total	62 (100.0)	55 (100.0)
χ^2^ = 0.436; P value = 0.509		

*Secondary headache is headache with a definitive and identifiable cause found for it i.e. those with pre-existing conditions that may cause the headache e.g. hypertension, cervical spondylosis, refractive error, sleep apnoea, malaria and other febrile conditions [15].

Most respondents reported ≤5 episodes of headache in the last 6 months (74.4%) and these were typically of short-lasting durations, <60 minutes (44.4%). There was no observed periodicity to the headaches in 57.3% of cases (see Table 3). Most of the headaches were not located in any particular part of the head or side-locked (71.7%); were described as mildly severe in 59.8% of cases while 88.0% of respondents did not suffer any sleep disruption. The headaches were often not significantly disabling (73.4%) and in 93.2% of respondents did not lead to absenteeism or affect productivity at work (Table 4).

**Table 3:** **Characterization of the headaches**

Variable/Characteristics	Frequency (N =117)	Percent
***Number of episodes in past 6 months***		
1 – 5	87	74.4
6 – 10	25	21.4
11 – 15	3	2.6
16 – 20	2	1.6
**Usual duration of headaches**		
Seconds	19	16.2
Minutes	52	44.4
Hours	36	30.9
Days	10	8.5
***Usual time of day of the headache***		
Morning	18	15.4
Afternoon	15	12.8
Night	13	11.1
Continuous	4	3.4
No particular time	67	57.3
***Is the headache becoming stronger, last longer or occur more frequent?***		
Yes	22	18.8
No	95	81.2
**What is the commonest nature of the headache?**		
Throbbing/exploding	43	36.8
Sharp	4	3.4
Tightness	5	4.3
Dull	6	5.1
Aching	24	20.5
Pressure in head	32	27.3
Grinding	3	2.6

**Table 4:** **Usual location and severity of the headache**

***Usual Location of headache***	Frequency	Percent
Left side	3	2.6
Forehead	9	7.7
Around the head/ill-defined	11	9.4
Right side	2	1.7
Both Temples	2	1.7
Top of the head	1	0.9
Neck	2	1.7
Back of head	3	2.6
No particular side	84	71.7
***Severity of headache***		
Mild	70	59.8
Moderate	45	38.5
severe	2	1.7
***Is the headache strong enough to wake you from sleep?***		
Yes	14	12.0
No	103	88.0
***Effect of headache on daily activities***		
No significant disability	16	13.7
Mild disability	86	73.4
Moderate	3	2.6
Severe disability	12	10.3
***Headache –related work absenteeism or reduced productivity?***		
Yes	8	6.8
No	109	93.2

**Figure 2:**
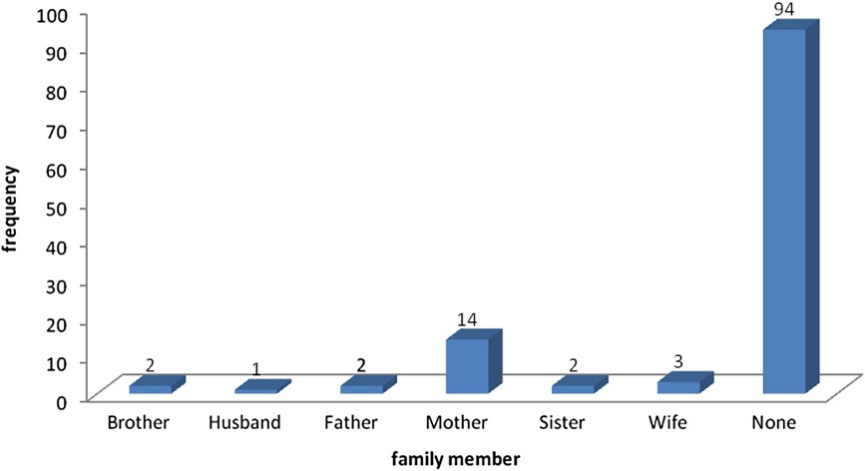
**Family member with history of headaches, migraines, sick headaches, motion sickness or had trouble taking birth control pills because of headaches.**

Stress (35.0%) and head trauma/illness/infection (18.8%) were the commonest predisposing conditions to the headache (Table 5). Refractive errors were present in 16.2% of respondents with headaches. In 25.6% there were headache prodromes and these included irritability (10.3%) and fatigue (5.1%). During the headaches, associated symptoms occurred in 30.8% of respondents and these included nasal congestion, redness of eyes, sinusitis or allergies (26.5%) as depicted in Table 6. In most cases, there was no known family history of migraines or other chronic headaches (Figure 2).

**Table 5:** **Predisposing conditions to the headache**

Factors preceding the headache	Frequency (N =117)	Percent
Accident, illness or infection	22	18.8
Odours	5	4.3
Fatigue	34	29.1
School	2	1.7
Hunger	17	14.5
Noise	4	3.4
Stress	41	35.0
Exercise	1	0.9
Family problem	2	1.7
Menstrual flow	2	1.7
Lack of sleep	8	6.8
Hot weather	2	1.7
None	49	41.9
**Existing chronic medical conditions that may cause headache**		
Hypertension	10	8.5
Cervical spondylosis	3	2.6
Refractive errors	19	16.2
Diabetes mellitus	2	1.7
Sleep apnoea	1	0.9
None	97	82.9

Note that some respondents filled more than one option.

**Table 6:** **Headache prodromes and other features associated with the headaches**

	Frequency (N =117)	Percent
***Presence of warning signs before headache***		
Yes	30	25.6
No	87	74.4
***Warning signs***		
Pallor	1	0.9
Mood swing	6	5.1
Irritability	12	10.3
Dizziness	3	2.6
Tired/sleepy	6	5.1
Rings around the eyes	1	0.9
Hyperactivity	1	0.9
Eye problems	2	1.7
None	104	88.9
Other symptoms associated with the headaches		
***Presence of other symptoms during the headaches***		
Yes	36	30.8
No	81	69.2
Nasal congestion, redness of eyes, sinusitis or allergies associated with the headache	31	26.5
Nausea.	2	1.7
Stomach pain	9	7.7
Vomiting	1	0.9
Confusion	3	2.6
Numbness in arms and legs	6	5.1
Diarrhoea	1	0.9
Dropping of the eyes	1	0.9
Fever	12	10.3

Note that some respondents filled more than one option.

Management of headache was varied among respondents. In most cases (47.9%) no intervention was required. However in other instances, investigations (11.1%) and eye checks (7.7%) were done. The over-the counter- available analgesic, paracetamol, (83.8%) was the commonest treatment received (Table 7).

**Table 7:** **Management received for the last headache episode**

Management actions	Frequency (N =117)	Percent
*Headache was managed by –*		
Health worker	19	16.2
Self	42	35.9
No treatment received	56	47.9
*A. Investigations done*		
Laboratory	13	11.1
Eye check	9	7.7
*B. Treatment received*		
Anti-malaria	7	6.0
Ergotamine	1	0.9
Food	1	0.9
Ibuprofen	5	4.3
Other NSAIDs*	2	1.7
Paracetamol	98	83.8
Tramadol (narcotic analgesic)	1	0.9
Eye glasses were prescribed	16	13.7
Relaxation	1	0.9
*Other actions that relieve the headaches*		
Cold compress	13	11.1
Eating	20	17.1
Massage	3	2.6
Moving around	3	2.6
Relaxation	47	40.2
Sleep	31	26.5
Vomiting	1	0.9
Others	1	0.9

*NSAIDs = non-steroidal anti-inflammatory agents.

Note that some respondents filled more than one option.

The health workers’ ages did not significantly affect both the presence and treatment of headache (p = 0.483 and 0.293 respectively) but significantly affected the type of headache (p = 0.005) i.e. whether it was primary or secondary headache (Table 8). Years of working in the hospital did not significantly affect the prevalence of headache (P = 0.123), type of headache (P = 0.423) or treatment of the headaches (P = 0.535) as shown in Table 9. There was no correlation between the number of headache episodes and the number of years worked in the hospital [Pearson Correlation (r) = - 0.066] or age of the health worker [r = 0.001].

**Table 8:** **Age group and management of headache**

	Age group in years					
Variables	15 -24	25 - 34	35 - 44	45 - 54	55 - 64	≤ 65
***Presence of headache***						
Yes	12(92.3)	53(85.5)	34(94.4)	13(86.7)	4(66.7)	1(100.0)
No	1(7.7)	9(14.5)	2(5.6)	2(23.3)	2(33.3)	0(0.0)
Total	13 (100.0)	62(100.0)	36(100.0)	15(100.0)	6(100.0)	1(100.0)
Likelihood-ratio χ^2^ = 4.480; P value = 0.483						
***Type of headache***						
Primary	7(58.3)	45(84.9)	27(79.4)	6(46.2)	1(33.3)	0(0.0)
Secondary	5(41.7)	8(15.1)	7(20.6)	7(53.8)	3(66.7)	1(100.0)
Total	12(100.0)	53(100.0)	34(100.0)	13(100.0)	4(100.0)	1(100.0)
Likelihood-ratio χ^2^ = 16.995; P value = 0.005 (significant)						
***Treatment of headache***						
Other health worker	10(83.3)	33(62.3)	18(52.9)	10(76.9)	3(66.7)	1(100.0)
Self	2(16.7)	20(37.7)	16(47.1)	3(23.1)	1(33.3)	0(0.0)
Total	12(100.0)	53(100.0)	34(100.0)	13(100.0)	4(100.0)	1(100.0)
Likelihood-ratio χ^2^ = 6.135; P value = 0.293

**Table 9:** **Number of years worked in the hospital and management of headache**

	Number of years worked in the hospital		
Variables	1 - 10	11 - 20	21 - 30
***Presence of headache***			
Yes	101(87.8)	11(100.0)	5(71.4)
No	14(12.2)	0(0.0)	2(28.6)
Total	115(100.0)	11(100.0)	7(100.0)
Likelihood-ratio χ^2^ = 4.199; P value =0.123			
***Type of headache***			
Primary	75(65.2)	8(72.7)	3(42.9)
Secondary	40(34.8)	3(27.3)	4(57.1)
Total	115(100.0)	11(100.0)	7(100.0)
Likelihood-ratio χ^2^ = 1.719; P value = 0.423			
***Treatment of headache***			
Other health worker	78(67.8)	7(63.6)	6(85.7)
Self	37(32.2)	4(36.4)	1(14.3)
Total	115(100.0)	11(100.0)	7(100.0)
Likelihood-ratio χ^2^ = 1.250; P value = 0.535			

## Discussion

Headache is the commonest presenting neurological disorder in most communities and clinical settings worldwide [2, 12]. Studies from Nigeria, including Enugu, also support this [1, 3, 13]. The prevalence of headache in health care workers has been variously reported from Western countries [16, 17] but there is a paucity of similar data from Nigeria and Africa. There was an inability to assess headaches as distinctly experienced in the various cadres of hospital workers and it was also not possible in this study to ascertain distinct headache entities and their roles. Other limitations of this study were its small sample size, the possibility of recall bias arising from patients’ answers to occurrences of headaches in the past 6 months, and use of a 3-point pain scale instead of the 10-point Visual Analogue Scale (VAS) which has greater scale refinement and discrimination power. VAS has been noted to be a valid instrument for measurement of pain intensity in patients with headaches [18].

A prevalence of 88.0% was obtained for headaches amongst the hospital workers, with slightly higher rates in females than males. Though the study periods vary, the figure compares favourably with the rate of 84.4% obtained amongst from health workers in the United States [16] but is significantly higher than the 54.7% and 27.1% prevalence rates obtained from Italian and Turkish health workers respectively [16, 19]. A survey of headache in Ethiopian textile workers found a prevalence of 73% [12]. The headache prevalence of 88% for hospital workers in this study compares favourably with the 88.3% prevalence found in a study of medical students in the same locality [20]. The prevalence is also higher than the community prevalence rates of 51% and 23.1% seen in Ibadan, South West Nigeria and rural south Tanzania respectively [11, 13]. It is possible that the different figures may reflect a combination of environmental challenges, durations of study and varied survey instruments used.

It is well noted that females tend to have higher rates for headache prevalence across cultures and continents [1, 3, 12, 16, 17, 19] and while this seemed to be the case in our study, the difference was not statistically significant. Reasons adduced for the higher female prevalence include the influence of oestrogens and progesterone on headaches after menarche and the greater propensity for females to seek medical attention for headaches [21].

Most of our subjects had probable primary headaches although no further attempts were made in this study to distinguish between the various different types (which include the trigeminal associated cephalalgias (TACs), migraine and tension- type headaches). Headaches were of short duration (<60 minutes) and were not side –locked in most instances unlike the longer duration (>6 hours) migraine headaches noted in the Turkish study [19]. Migraine headache prevalence rate is uniformly low across much of Africa but was found to be significantly high in a cohort of textile mill workers in Ethiopia [12, 22].

Stress, probably related to challenges in the work environment, played the greatest role (35.0%) in this study and this reflected in the calming role attributed to relaxation techniques utilized by the health workers (40.2%) to manage their headaches. Besides life and work stress, personality traits such as aggression, anger and type A behaviour are factors that may aggravate stress and are frequently found in headache patients but were not sought for in this study [22–24].

There was no significant association or correlation found between the prevalence of headaches and years of working experience in this study. Non-pharmacological treatment was suitable for almost half of respondents (47.9%) while the over-the –counter medicine, paracetamol, was the most utilised drug treatment. This finding is essentially similar to that of health workers with headaches in the Unites States but contrasts with the use of NSAIDs in a Turkey study [17, 19]. Despite working in a health facility, self-medication was commonly practised (35.9%) but this was even more significant among Turkish health workers (54.6%) [19].

The low rate of medical consultation for headache in hospital workers is of interest. In this study centre, headache ranks low among the disorders seen at both the Pain Clinic and Neurology Clinic accounting for only 2.7% of all neurological cases seen in the latter and 9^th^ of the top 10 disorders encountered [3]. Some reasons adduced for the low rate of presentations to clinics for headaches as well as low rates of success in headache treatment amongst Africans include underdiagnoses or misdiagnoses due to lack of adequate knowledge by healthcare professionals, headache sufferers being ignorant of effective prophylaxis and treatment, perception of headaches as a trivial problem, and great tolerance to pain [25–29]. Other reasons include poor healthcare facilities [30], low economic power [25], gender/child discrimination [28], and unavailability of effective medication [28, 29]. The authors are of the opinion that African patients’ preference for/reliance on non-drug options (complementary and alternative medicine, CAM) [25, 28, 30] for pain relief may also be contributory.

Of important economic interest is the rarity of absenteeism from work or loss of productive time as reported in this study. These factors are important because many headache sufferers are at the peak of their work-productive life [26]. Employers may lose an average of 12 days per year because of an employee headache syndrome [27]. The authors relate reason for the rarity of work absenteeism and loss of productive time to the majority of headaches being of a mild nature with low disabling rates. A similar negligible rate of absenteeism was the outcome among Italian health workers with headache [16].

## Conclusion

This preliminary study has revealed headaches to be common in this community of healthcare workers. However, the seemingly low effect of headache on health workers productivity in this study, despite its high prevalence rate and contrary to views [28] from other African studies, is of notable relief in a developing economy like Nigeria where health indicators are unimpressive and medical services still face huge challenges. In addition, presentation to Pain or Neurology clinic for headache disorders by respondents in this study has been shown to be low, demonstrating the need for increased and continuous health awareness on headache disorders as well as enhanced occupational health services in Nigerian hospitals. By the findings of this work, the authors encourage more robust studies on headache disorders among healthcare workers in African countries with a view to informing better practice decisions and reducing the global headache burden.

## References

[1] Onwuekwe IO, Ezeala-Adikaibe B, Ekenze OS (2012). Neurological disease burden in two semi-urban communities in South East Nigeria. Nig J Med.

[2] Scher AL, Stewart WF, Lipton RB, Crombie IK, Croft PR, Linton SJ (1999). Migraine and headache: a meta-analytic approach. Epidemiology of Pain.

[3] Onwuekwe IO, Ezeala-Adikaibe B (2011). Prevalence and distribution of neurological disease in a Neurology Clinic in Enugu, Nigeria. Annals Med Health Science Res.

[4] Vas J, Rebollo A, Perea-Milla E, Mendez C, Font C, Gomez-Rio M, Martin-Avila M, Cabrera-Iboleon J, Caballero MD, Olmos MA, Aguilar I, Faus V, Martos F (2008). Study protocol for a pragmatic randomised controlled trial in general practice investigating the effectiveness of acupuncture against migraine. BMC Compl Alternative Med.

[5] Fowler TJ, Scadding JW, Fowler TJ, Scadding JE (2003). Introduction. Clinical Neurology.

[6] Manack AN, Buse DC, Lipton RB (2011). Chronic migraine: epidemiology and disease burden. Curr Pain Headache Rep.

[7] World Health Organisation (WHO) (2014). Headache Disorders.

[8] Pinder RM (2006). Migraine – a suitable case for treatment?. Neuropsychiatr Dis Treat.

[9] Patel V, Simbine AP, Soares IC, Weiss HA, Wheeler E (2007). Prevalence of severe mental and neurological disorders in Mozambique: a population-based survey. Lancet.

[10] Hu H, Markson LE, Lipton RB, Stewart WF, Berger ML (1999). Burden of migraine in the United States: disability and economic costs. Arch Intern Med.

[11] Dent W, Spiss HK, Helbok R, Matuja WBP, Sheunemann S, Schmutzard E (2004). Prevalence of migraine in a rural area in South Tanzania: a door-to-door survey. Cephalalgia.

[12] Takele GM, Haimanot RT, Martelleti P (2008). Prevalence and burden of headache in Akaki Textile Mill Workers, Ethiopia. J Headache Pain.

[13] Osuntokun BO, Adeuja AO, Nottidge VA, Bademosi O, Olumide AO, Ige O, Yaria F, Schoenberg BS, Bolis CL (1992). Prevalence of headache and migrainous headache in Nigerian Africans: a community-based study. East Afr Med J.

[14] Owolabi LF, Gwaram B (2012). Clinical Profile of Primary Headache disorders in Kano, Northwestern Nigeria. J Med Tropics.

[15] ICHD (2013). International classification of headache disorders (ICHD) 3rd edition (beta version). Cephalalgia.

[16] Hughes MD, Wu J, Williams TC, Loberger JM, Hudson MF, Burdine JR, Wagner PJ (2013). The experience of headaches in health care workers: opportunity for care improvement. Headache.

[17] Sokolovic E, Riederer F, Szucs T, Agosti R, Sandor PS (2013). Self-reported headache among the employees of a Swiss University Hospital: prevalence, disability, current treatment and economic impact. J Headache Pain.

[18] Lundqvist C, Benth JS, Grande RB, Aaseth K, Russel MB (2009). A vertical VAS is a valid instrument for monitoring headache pain intensity. Cephalalgia.

[19] Dikici S, Baltaci D, Arslan G, Atar G, Ercan N, Yilmaz A, Kara IH (2013). Headache frequency among the health care workers and the relationship working conditions. Abant Med J.

[20] Ezeala-Adikaibe B, Ekenze OS, Onwuekwe IO, Ulasi II (2012). Frequency and pattern of headache among medical students at Enugu, South East Nigeria. Nig J Med.

[21] Martin BC, Dorfman JH, McMillan CA (1994). Prevalence of migraine headache and association with sex, age, race, and rural/urban residence: a population-based study of Georgia Medicaid recipients. Clin Ther.

[22] Sjösten N, Nabi H, Westerlund H, Singh-Manoux A, Dartigues J, Goldberg M, Zins M, Oksanen T, Salo P, Pentti J, Kivimäki M, Vahtera J (2011). Influence of retirement and work stress on headache prevalence: a longitudinal modelling study from the GAZEL cohort. Cephalalgia.

[23] Fichera LV, Andreassi JL (1998). Stress and personality as factors in women’s cardiovascular reactivity. Int J Psychophysiol.

[24] Abbate-Daga G, Fassino S, Lo Giudice R, Rainero I, Gramaglia C, Marech L, Amianto F, Gentile S, Pinessi L (2007). Anger, depression and personality dimensions in patients with migraine without aura. Psychother Psychosom.

[25] Haimanot RT (2003). Burden of headache in Africa. J Headache Pain.

[26] Vinding G, Zeeberg P, Lyngberg A, Nielsen R, Jensen R (2007). The burden of headache in a patient population from a specialized headache centre. Cephalalgia.

[27] Von Korff M, Stewart WF, Simon D, Lipton RB (1998). Migraine and reduced work performance: A population-based diary study. Neurology.

[28] Mengistu G, Alemayehu S (2013). Prevalence and burden of primary headache disorders among a local community in Addis Ababa, Ethiopia. J Headache Pain.

[29] Takele GM, Tekle Haimanot R, Martelletti P (2008). Prevalence and burden of primary headache in Akaki textile mill workers, Ethiopia. J Headache Pain.

[30] Haimanot RT, Martelletti P, Steiner T (2011). Headache in the Tropics: Sub-Saharan Africa. Handbook of Headache: Practical Management.

